# (1*S*,2*S*)-2-[(*S*)-2,2,2-Tri­fluoro-1-hy­droxy­eth­yl]-1-tetra­lol

**DOI:** 10.1107/S2414314623002171

**Published:** 2023-03-15

**Authors:** Kristian Radan, Andrej Emanuel Cotman, Matic Lozinšek

**Affiliations:** a Department of Inorganic Chemistry and Technology, Jožef Stefan Institute, Jamova cesta 39, 1000 Ljubljana, Slovenia; b Department of Pharmaceutical Chemistry, Faculty of Pharmacy, University of Ljubljana, Aškerceva cesta 7, 1000 Ljubljana, Slovenia; Sunway University, Malaysia

**Keywords:** tetra­lol, crystal structure, asymmetric transfer hydrogenation, tri­fluoro­methyl group

## Abstract

The crystal structure of the title enanti­opure tetralol derivative {systematic name: (1*S*,2*S*)-2-[(*S*)-2,2,2-tri­fluoro-1-hy­droxy­eth­yl]-1,2,3,4-tetra­hydro­naph­thalen-1-ol}, C_12_H_13_F_3_O_2_, synthesized by asymmetric transfer hydrogenation, was elucidated by low-temperature single-crystal X-ray diffraction. The enanti­opure compound crystallizes in the Sohncke space group *P*2_1_2_1_2_1_ with one mol­ecule in the asymmetric unit and features intra­molecular as well as inter­molecular O—H⋯O hydrogen bonding. The absolute configuration was established from anomalous dispersion effects.

## Abstract 2

The title enanti­opure tetra­lol derivative synthesized by asymmetric transfer hydrogenation crystallizes in the Sohncke space group *P*2_1_2_1_2_1_ and features one mol­ecule in the asymmetric unit. In the crystal, mol­ecules are hydrogen-bonded into chains running parallel to [010].

## Graphical abstract

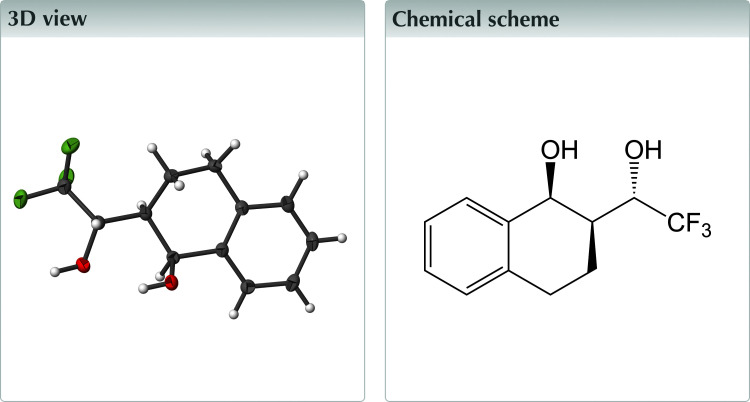

## Structure description

**Figure 1:**
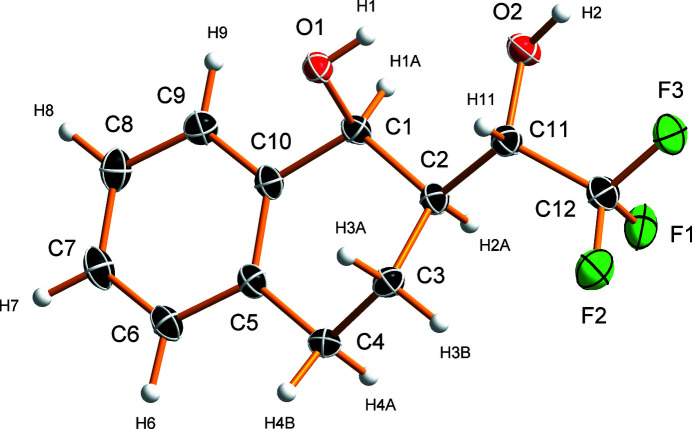
Mol­ecular structure of (1*S*,2*S*)-2-[(*S*)-2,2,2-tri­fluoro-1-hy­droxy­eth­yl]-1-tetra­lol showing the atom-labeling scheme. Thermal displacement ellipsoids are drawn at the 50% probability level and the hydrogen atoms are shown as spheres of arbitrary radius.

Homochiral fluorinated alcohols, which are considered to be emerging structural motifs in medicinal chemistry (Cotman, 2021 ▸), can be obtained in high yields employing dynamic kinetic resolution (DKR) with Noyori–Ikariya asymmetric transfer hydrogenation (ATH) (Betancourt *et al.*, 2021 ▸; Cotman *et al.* 2022 ▸; Molina Betancourt *et al.*, 2022 ▸). When Ru^II^-catalyzed DKR–ATH was applied to CF_3_CO-substituted benzofused cyclic ketones, it was observed that single or double reduction occurs, yielding either diastereo- and enanti­o­pure monoalcohols or 1,3-diols (Cotman *et al.*, 2016 ▸). The crystal structure of the mono-reduced product (*S*)-2-[(*S*)-2,2,2-tri­fluoro-1-hy­droxy­eth­yl]-1-tetra­lone has been described previously (Motaln *et al.*, 2023 ▸) and herein the crystal structure of the corres­ponding diol is presented.

(1*S*,2*S*)-2-[(*S*)-2,2,2-Tri­fluoro-1-hy­droxy­eth­yl]-1-tetra­lol crystallizes in the ortho­rhom­bic *P*2_1_2_1_2_1_ space group with one mol­ecule in the asymmetric unit (Fig. 1 ▸). The cyclo­hexa­nol ring adopts a half-chair conformation (Cremer & Pople, 1975 ▸), with the C2 atom located 0.251 (3) Å below and the C3 atom 0.497 (4) Å above the plane defined by atoms C1, C4, C5, and C10 (r.m.s.d. of 0.013 Å). This plane is essentially coplanar with the aromatic ring – the angle between the plane normals is 2.79 (9)° and the r.m.s. deviation of the plane defined by all coplanar atoms C1, C4–C10 is 0.025 Å. Tetra­lol derivatives with similar half-chair conformations have been reported, for example, 2,2,2-tri­fluoro-*N*-(1-hy­droxy-1,2,3,4-tetra­hydro­naphthalen-2-yl)acetamide (CSD refcode ALUXUC; Miyazawa *et al.*, 2016 ▸), (1*S*,2*S*)-7-meth­oxy-2-(tri­fluoro­meth­yl)-1-tetra­lol (YEDBOC; Cotman *et al.*, 2022 ▸), and plastically flexible (1*R*,2*S*)-2-(tri­fluoro­methyl­thio)-1-tetra­lol (YEDCAP; Cot­man *et al.*, 2022 ▸).

**Figure 2:**
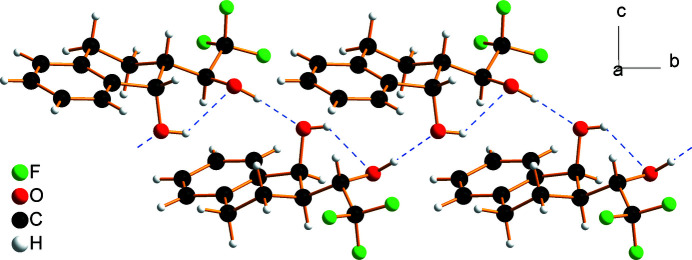
Helical hydrogen-bonded chain extending parallel to [010] that involves inter­molecular and intra­molecular O—H⋯O hydrogen bonds, which are indicated by blue dashed lines.

In the crystal of the title compound, intra­molecular and inter­molecular O—H⋯O hydrogen bonds with O⋯O distances of 2.854 (2) and 2.789 (2) Å, respectively, link adjacent mol­ecules related by the 2_1_ screw axis, into chains parallel to [010] (Table 1 ▸ and Figs. 2 ▸ and 3 ▸). The graph-set motifs of the hydrogen bonds are *S*(6) and *C*(6) (Etter *et al.*, 1990 ▸).

## Synthesis and crystallization

The title compound was prepared from 2-tri­fluoro­acetyl-1-tetra­lone (100 mg, 0.412 mmol) added to a HCO_2_H/Et_3_N 3:2 (0.21 ml) solution containing the active (*S*,*S*)-di­phenyl­ethyl­enedi­amine-based Ru^II^ catalyst with an S/C ratio of 1000:1 (Cotman *et al.*, 2016 ▸). Upon addition of the co-solvent chloro­benzene (0.55 ml), the mixture was warmed to 60 °C and stirred for 24 h, while being continuously flushed with N_2_. The resulting mixture was partitioned between EtOAc (10 ml) and H_2_O (5 ml), with the organic layer later washed with H_2_O (5 ml) and brine (5 ml), filtered through a bed of silica gel/Na_2_SO_4_, and concentrated. The crude product was recrystallized from a 5:1 mixture of petroleum ether and diethyl ether affording colorless prisms (37 mg; 36% yield; diastereomeric ratio 97:3:0:0; enanti­omeric excess >99.9%). A suitable crystal was selected under a polarizing microscope and attached to a MiTeGen Dual Thickness MicroLoop using Baysilone-Paste (Bayer-Silicone, mittelviskos) as the adhesive.

## Refinement

The crystal data, data collection, and structure refinement details are summarized in Table 2 ▸. The positions of the hydrogen atoms and their isotropic displacement parameter *U* were freely refined (Cooper *et al.*, 2010 ▸). The absolute configuration was established as *S*,*S*,*S* for C1, C2, and C11, respectively, based on anomalous dispersion effects [Flack *x* = 0.06 (5); Hooft *y* = 0.07 (3)] (Parsons *et al.*, 2013 ▸; Hooft *et al.*, 2008 ▸).

## Supplementary Material

Crystal structure: contains datablock(s) I. DOI: 10.1107/S2414314623002171/tk4089sup1.cif

Structure factors: contains datablock(s) I. DOI: 10.1107/S2414314623002171/tk4089Isup2.hkl

Supporting information file. DOI: 10.1107/S2414314623002171/tk4089Isup3.cml

CCDC reference: 2246795

Additional supporting information:  crystallographic information; 3D view; checkCIF report

## Figures and Tables

**Figure 3:**
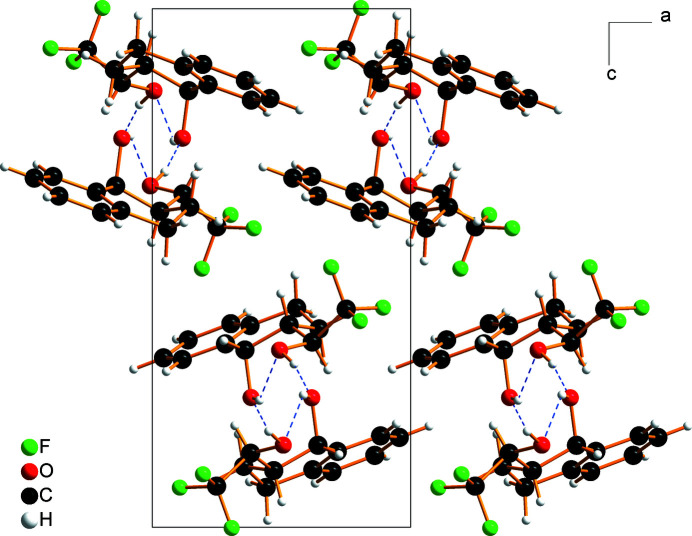
Mol­ecular packing of the title compound viewed along [010]. The helical hydrogen-bonded chains are shown by blue dashed lines.

**Table 1:** Hydrogen-bond geometry (Å, °)

*D*—H⋯*A*	*D*—H	H⋯*A*	*D*⋯*A*	*D*—H⋯*A*
O1—H1⋯O2	0.83 (4)	2.23 (4)	2.854 (2)	133 (3)
O2—H2⋯O1^i^	0.94 (4)	1.87 (4)	2.789 (2)	168 (3)

Symmetry code: (i) 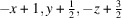 .

**Table 2:** Experimental details

Crystal data	
Chemical formula	C_12_H_13_F_3_O_2_
*M*_r_	246.22
Crystal system, space group	Orthorhombic, *P*2_1_2_1_2_1_
Temperature (K)	100
*a*, *b*, *c* (Å)	7.75558 (10), 9.02843 (10), 15.5656 (2)
*V* (Å^3^)	1089.92 (2)
*Z*	4
Radiation type	Cu *K*α
μ (mm^−1^)	1.17
Crystal size (mm)	0.08 × 0.07 × 0.06

Data collection	
Diffractometer	XtaLAB Synergy-S, Dualflex, Eiger2 R CdTe 1M
Absorption correction	Gaussian (*CrysAlis PRO*; Rigaku OD, 2022 ▸)
*T*_min_, *T*_max_	0.886, 1.000
No. of measured, independent and observed [*I* > 2σ(*I*)] reflections	38720, 2273, 2255
*R*_int_	0.053
(sin θ/λ)_max_ (Å^−1^)	0.630

Refinement	
*R*[*F* ^2^ > 2σ(*F* ^2^)], *wR*(*F* ^2^), *S*	0.029, 0.073, 1.06
No. of reflections	2273
No. of parameters	207
H-atom treatment	All H-atom parameters refined
Δρ_max_, Δρ_min_ (e Å^−3^)	0.35, −0.17
Absolute structure	Flack *x* determined using 929 quotients [(*I* ^+^)−(*I* ^−^)]/[(*I* ^+^)+(*I* ^−^)] (Parsons *et al.*, 2013 ▸)
Absolute structure parameter	0.06 (5)

Computer programs: *CrysAlis PRO* (Rigaku OD, 2022 ▸), *OLEX2.solve* (Dolomanov *et al.*, 2009 ▸), *SHELXL* (Sheldrick, 2015 ▸), *OLEX2* (Dolomanov *et al.*, 2009 ▸), *DIAMOND* (Brandenburg, 2005 ▸) and *publCIF* (Westrip, 2010 ▸).

## full crystallographic data

### Crystal data

**Table:**

C_12_H_13_F_3_O_2_	*D*_x_ = 1.501 Mg m^−^^3^
*M**_r_* = 246.22	Cu *K*α radiation, λ = 1.54184 Å
Orthorhombic, *P*2_1_2_1_2_1_	Cell parameters from 29238 reflections
*a* = 7.75558 (10) Å	θ = 2.9–75.7°
*b* = 9.02843 (10) Å	µ = 1.17 mm^−^^1^
*c* = 15.5656 (2) Å	*T* = 100 K
*V* = 1089.92 (2) Å^3^	Cube, colourless
*Z* = 4	0.08 × 0.07 × 0.06 mm
*F*(000) = 512	

### Data collection

**Table:**

XtaLAB Synergy-S, Dualflex, Eiger2 R CdTe 1M diffractometer	2273 independent reflections
Radiation source: micro-focus sealed X-ray tube, PhotonJet (Cu) X-ray Source	2255 reflections with *I* > 2σ(*I*)
Mirror monochromator	*R*_int_ = 0.053
Detector resolution: 13.3333 pixels mm^-1^	θ_max_ = 76.1°, θ_min_ = 5.7°
ω scans	*h* = −9→8
Absorption correction: gaussian (CrysAlisPro; Rigaku OD, 2022)	*k* = −11→11
*T*_min_ = 0.886, *T*_max_ = 1.000	*l* = −19→19
38720 measured reflections	

### Refinement

**Table:**

Refinement on *F*^2^	All H-atom parameters refined
Least-squares matrix: full	*w* = 1/[σ^2^(*F*_o_^2^) + (0.0325*P*)^2^ + 0.4546*P*] where *P* = (*F*_o_^2^ + 2*F*_c_^2^)/3
*R*[*F*^2^ > 2σ(*F*^2^)] = 0.029	(Δ/σ)_max_ < 0.001
*wR*(*F*^2^) = 0.073	Δρ_max_ = 0.35 e Å^−^^3^
*S* = 1.06	Δρ_min_ = −0.17 e Å^−^^3^
2273 reflections	Extinction correction: SHELXL-2019/2 (Sheldrick, 2015), Fc^*^=kFc[1+0.001xFc^2^λ^3^/sin(2θ)]^-1/4^
207 parameters	Extinction coefficient: 0.0016 (4)
0 restraints	Absolute structure: Flack *x* determined using 929 quotients [(*I*^+^)–(*I*^–^)]/[(*I*^+^)+(*I*^–^)] (Parsons *et al.*, 2013)
Primary atom site location: iterative	Absolute structure parameter: 0.06 (5)
Hydrogen site location: difference Fourier map	

### Special details

**Table:**

Geometry. All esds (except the esd in the dihedral angle between two l.s. planes) are estimated using the full covariance matrix. The cell esds are taken into account individually in the estimation of esds in distances, angles and torsion angles; correlations between esds in cell parameters are only used when they are defined by crystal symmetry. An approximate (isotropic) treatment of cell esds is used for estimating esds involving l.s. planes.

### Fractional atomic coordinates and isotropic or equivalent isotropic displacement parameters (Å^2^)

**Table:**

	*x*	*y*	*z*	*U*_iso_*/*U*_eq_	
F3	0.80337 (19)	0.85568 (15)	0.60230 (9)	0.0345 (3)	
F1	0.69235 (19)	0.72956 (16)	0.49867 (9)	0.0322 (3)	
O2	0.49295 (19)	0.76829 (16)	0.65842 (10)	0.0237 (3)	
F2	0.89287 (17)	0.63500 (16)	0.57649 (10)	0.0355 (4)	
O1	0.38269 (19)	0.51110 (16)	0.74976 (9)	0.0198 (3)	
C5	0.3786 (3)	0.2240 (2)	0.61093 (12)	0.0179 (4)	
C1	0.3588 (2)	0.4939 (2)	0.65812 (12)	0.0171 (4)	
C10	0.2838 (3)	0.3406 (2)	0.64639 (12)	0.0178 (4)	
C12	0.7506 (3)	0.7194 (2)	0.57933 (14)	0.0248 (5)	
C4	0.5590 (3)	0.2478 (2)	0.57692 (13)	0.0203 (4)	
C11	0.6192 (3)	0.6587 (2)	0.64276 (13)	0.0201 (4)	
C9	0.1166 (3)	0.3148 (2)	0.67633 (14)	0.0211 (4)	
C8	0.0440 (3)	0.1739 (3)	0.67329 (14)	0.0243 (4)	
C3	0.6458 (3)	0.3820 (2)	0.61755 (13)	0.0196 (4)	
C7	0.1391 (3)	0.0578 (2)	0.63868 (14)	0.0239 (4)	
C2	0.5296 (2)	0.5175 (2)	0.60927 (13)	0.0178 (4)	
C6	0.3043 (3)	0.0827 (2)	0.60742 (13)	0.0212 (4)	
H11	0.680 (4)	0.646 (3)	0.6961 (16)	0.025 (7)*	
H6	0.369 (3)	−0.005 (3)	0.5850 (16)	0.024 (6)*	
H9	0.055 (4)	0.395 (3)	0.6995 (17)	0.028 (7)*	
H8	−0.071 (4)	0.156 (3)	0.6950 (17)	0.031 (7)*	
H3A	0.666 (3)	0.359 (3)	0.6794 (16)	0.023 (6)*	
H1A	0.277 (3)	0.570 (3)	0.6402 (15)	0.015 (5)*	
H4A	0.553 (4)	0.266 (3)	0.5136 (17)	0.029 (7)*	
H2A	0.498 (3)	0.534 (3)	0.5494 (16)	0.022 (6)*	
H4B	0.623 (3)	0.155 (3)	0.5878 (17)	0.029 (7)*	
H7	0.094 (4)	−0.037 (3)	0.6371 (18)	0.031 (7)*	
H3B	0.757 (3)	0.401 (3)	0.5896 (17)	0.024 (6)*	
H1	0.415 (4)	0.598 (4)	0.755 (2)	0.053 (10)*	
H2	0.549 (5)	0.848 (4)	0.685 (2)	0.059 (10)*	

### Atomic displacement parameters (Å^2^)

**Table:**

	*U*^11^	*U*^22^	*U*^33^	*U*^12^	*U*^13^	*U*^23^
F3	0.0359 (7)	0.0259 (7)	0.0418 (7)	−0.0144 (6)	0.0096 (6)	−0.0073 (6)
F1	0.0371 (7)	0.0326 (7)	0.0271 (6)	−0.0095 (6)	0.0025 (6)	0.0059 (5)
O2	0.0221 (7)	0.0175 (7)	0.0314 (8)	0.0003 (6)	0.0003 (6)	−0.0025 (6)
F2	0.0200 (6)	0.0358 (8)	0.0507 (8)	−0.0024 (6)	0.0095 (6)	0.0009 (6)
O1	0.0238 (7)	0.0168 (7)	0.0189 (7)	−0.0023 (6)	0.0006 (6)	−0.0005 (6)
C5	0.0196 (9)	0.0172 (9)	0.0170 (8)	0.0007 (8)	−0.0039 (8)	0.0016 (7)
C1	0.0167 (9)	0.0158 (8)	0.0187 (9)	0.0013 (8)	−0.0009 (7)	−0.0002 (7)
C10	0.0180 (9)	0.0169 (9)	0.0184 (8)	0.0001 (8)	−0.0029 (7)	0.0025 (7)
C12	0.0261 (11)	0.0212 (10)	0.0273 (10)	−0.0053 (8)	0.0028 (8)	−0.0042 (8)
C4	0.0203 (10)	0.0178 (9)	0.0230 (10)	0.0025 (7)	0.0034 (8)	−0.0008 (8)
C11	0.0192 (9)	0.0181 (9)	0.0230 (9)	−0.0007 (8)	0.0010 (8)	−0.0015 (8)
C9	0.0178 (9)	0.0218 (10)	0.0236 (9)	0.0006 (8)	−0.0016 (8)	0.0010 (8)
C8	0.0186 (10)	0.0270 (10)	0.0273 (10)	−0.0064 (8)	−0.0037 (8)	0.0050 (9)
C3	0.0155 (8)	0.0211 (9)	0.0222 (9)	0.0006 (7)	0.0017 (8)	−0.0002 (8)
C7	0.0262 (11)	0.0189 (10)	0.0267 (10)	−0.0067 (8)	−0.0090 (9)	0.0023 (8)
C2	0.0180 (9)	0.0163 (8)	0.0192 (9)	−0.0014 (7)	0.0001 (8)	−0.0005 (7)
C6	0.0258 (10)	0.0169 (9)	0.0207 (9)	−0.0003 (8)	−0.0053 (8)	0.0010 (8)

### Geometric parameters (Å, º)

**Table:**

F3—C12	1.345 (2)	C4—H4A	1.00 (3)
F1—C12	1.338 (3)	C4—H4B	0.99 (3)
O2—C11	1.413 (2)	C11—C2	1.542 (3)
O2—H2	0.94 (4)	C11—H11	0.96 (3)
F2—C12	1.342 (3)	C9—C8	1.392 (3)
O1—C1	1.447 (2)	C9—H9	0.94 (3)
O1—H1	0.83 (4)	C8—C7	1.390 (3)
C5—C10	1.398 (3)	C8—H8	0.96 (3)
C5—C4	1.511 (3)	C3—C2	1.525 (3)
C5—C6	1.401 (3)	C3—H3A	1.00 (3)
C1—C10	1.512 (3)	C3—H3B	0.98 (3)
C1—C2	1.542 (3)	C7—C6	1.389 (3)
C1—H1A	0.98 (2)	C7—H7	0.93 (3)
C10—C9	1.397 (3)	C2—H2A	0.98 (3)
C12—C11	1.522 (3)	C6—H6	1.00 (3)
C4—C3	1.524 (3)		
			
C11—O2—H2	107 (2)	O2—C11—H11	106.0 (16)
C1—O1—H1	103 (2)	C12—C11—C2	112.35 (17)
C10—C5—C4	121.21 (17)	C12—C11—H11	105.9 (16)
C10—C5—C6	119.02 (18)	C2—C11—H11	114.5 (17)
C6—C5—C4	119.76 (18)	C10—C9—H9	117.8 (17)
O1—C1—C10	105.47 (15)	C8—C9—C10	121.10 (19)
O1—C1—C2	111.18 (15)	C8—C9—H9	121.1 (17)
O1—C1—H1A	106.8 (14)	C9—C8—H8	120.9 (17)
C10—C1—C2	113.45 (16)	C7—C8—C9	119.2 (2)
C10—C1—H1A	111.3 (14)	C7—C8—H8	119.9 (17)
C2—C1—H1A	108.5 (14)	C4—C3—C2	110.02 (16)
C5—C10—C1	122.30 (17)	C4—C3—H3A	107.8 (16)
C9—C10—C5	119.60 (18)	C4—C3—H3B	110.2 (15)
C9—C10—C1	118.03 (17)	C2—C3—H3A	110.1 (15)
F3—C12—C11	111.16 (18)	C2—C3—H3B	109.8 (15)
F1—C12—F3	106.85 (18)	H3A—C3—H3B	109 (2)
F1—C12—F2	106.60 (18)	C8—C7—H7	120.7 (17)
F1—C12—C11	114.03 (18)	C6—C7—C8	120.20 (19)
F2—C12—F3	106.17 (17)	C6—C7—H7	119.1 (17)
F2—C12—C11	111.57 (18)	C1—C2—C11	109.55 (16)
C5—C4—C3	112.12 (16)	C1—C2—H2A	105.9 (15)
C5—C4—H4A	109.0 (16)	C11—C2—H2A	108.1 (15)
C5—C4—H4B	106.6 (16)	C3—C2—C1	110.76 (16)
C3—C4—H4A	107.5 (16)	C3—C2—C11	111.62 (16)
C3—C4—H4B	112.4 (16)	C3—C2—H2A	110.7 (15)
H4A—C4—H4B	109 (2)	C5—C6—H6	121.9 (14)
O2—C11—C12	108.89 (16)	C7—C6—C5	120.86 (19)
O2—C11—C2	108.96 (16)	C7—C6—H6	117.2 (14)
			
F3—C12—C11—O2	47.8 (2)	C10—C1—C2—C11	−165.55 (16)
F3—C12—C11—C2	168.61 (17)	C10—C1—C2—C3	−42.0 (2)
F1—C12—C11—O2	−73.1 (2)	C10—C9—C8—C7	−0.9 (3)
F1—C12—C11—C2	47.8 (2)	C12—C11—C2—C1	−159.85 (17)
O2—C11—C2—C1	−39.1 (2)	C12—C11—C2—C3	77.1 (2)
O2—C11—C2—C3	−162.14 (16)	C4—C5—C10—C1	−4.0 (3)
F2—C12—C11—O2	166.11 (16)	C4—C5—C10—C9	179.31 (17)
F2—C12—C11—C2	−73.1 (2)	C4—C5—C6—C7	179.64 (17)
O1—C1—C10—C5	−108.6 (2)	C4—C3—C2—C1	62.5 (2)
O1—C1—C10—C9	68.1 (2)	C4—C3—C2—C11	−175.18 (16)
O1—C1—C2—C11	−46.9 (2)	C9—C8—C7—C6	−0.1 (3)
O1—C1—C2—C3	76.7 (2)	C8—C7—C6—C5	0.7 (3)
C5—C10—C9—C8	1.5 (3)	C2—C1—C10—C5	13.3 (3)
C5—C4—C3—C2	−52.3 (2)	C2—C1—C10—C9	−169.99 (17)
C1—C10—C9—C8	−175.33 (19)	C6—C5—C10—C1	175.72 (17)
C10—C5—C4—C3	23.6 (3)	C6—C5—C10—C9	−1.0 (3)
C10—C5—C6—C7	−0.1 (3)	C6—C5—C4—C3	−156.10 (18)

### Hydrogen-bond geometry (Å, º)

**Table:**

*D*—H···*A*	*D*—H	H···*A*	*D*···*A*	*D*—H···*A*
O1—H1···O2	0.83 (4)	2.23 (4)	2.854 (2)	133 (3)
O2—H2···O1^i^	0.94 (4)	1.87 (4)	2.789 (2)	168 (3)

Symmetry code: (i) −*x*+1, *y*+1/2, −*z*+3/2.
